# Commentary on: Applying Principles of Evolutionary Biology to Plastic Surgery at an Organizational Level Predicts an Extinction Event

**DOI:** 10.1093/asjof/ojad075

**Published:** 2023-08-12

**Authors:** Keith Brandt

See the Original Article here.

The authors of the article, Applying Principles of Evolutionary Biology to Plastic Surgery at an Organizational Level Predicts an Extinction Event,^1^ should be congratulated for tackling such a difficult and misunderstood process as medical licensure and certification. Their use of a “sociobiologic perspective,” however, to compare medical organization to animal taxonomy and evolutionary biology confuses an already difficult subject. While it may be interesting to view organized medicine divided into domains, phyla, and species, a more pragmatic view is needed to deal with 21st century reality.

The authors correctly point out that state medical licenses are not specialty specific, and that once granted a license, physicians can practice in any area of medicine they choose, regardless of their training. This is exactly why the system of specialty boards was created. Who better than the specialists themselves can identify the individuals who are properly trained to practice in that specialty. Patients, insurance companies, and hospital credentialing committees depend on the specialty boards to “self” regulate their individual specialty. This requires the boards to assess an individual's knowledge and skill before certifying and to continuously monitor certified diplomates to document adequate knowledge and skill throughout their career. If individuals fall below the standard of a specialist, then the certification must be revoked. The willingness to revoke a certificate is the most difficult of the self-regulation requirements; however, the one that creates the most legitimacy. Unfortunately, loss of certification does not necessarily lead to loss of licensure or even the cessation of practicing in that specialty. Only a loss of licensure completely prevents a physician from practicing in that state. In the United States, 20% of practicing physicians are not certified.

The authors argue that American Board of Medical Specialties (ABMS) Member Boards, like the American Board of Plastic Surgery (ABPS), often add additional requirements such as hospital privileges and working in an accredited surgery center which “adds an additional layer of policing for those practitioners who elect to join…thereby subjecting practitioners to further regulatory scrutiny.” ABMS Member Boards prioritize patient safety which is why patients, hospitals, and insurance companies look to ABMS board certification, to identify safe, knowledgeable, and skilled practitioners.

Deserving or not, the specialty of plastic surgery has a public image problem. Physicians from other ABMS boards, noncertified physicians, and even nonphysicians constantly encroach on the practice of plastic surgery, both cosmetic and reconstructive. The only way to combat this is to hold ABPS Diplomates to the higher ABMS standards and maintain respected self-regulation. Accordingly, the ABPS requires hospital privileges so that surgeons have a higher level facility to admit their patients to, for advanced care, when required. ABPS requires accreditation of surgery centers and office OR facilities to ensure that diplomates practice in an environment that has the equipment and trained personnel to manage patients safely. Yes, it is “further regulatory scrutiny,” but one ABPS believes is essential to upholding the highest standard of care in the specialty.

Within the article, the authors devote 3 pages describing various controversies among boards within and outside the ABMS community. These are all real-life examples of how by maintaining the higher ABMS standards, ABPS has weathered the crisis and remains the most respected moniker within the field of plastic surgery. Despite all the negativity expressed in the article about the ABMS system and the author's prediction that this will lead to its “extinction,” ABMS and its Member Boards remain the highest standard for physician certification.

Near the end of the article, the authors propose a solution to avoid all this competition by creating a new specialty “Restorative Healthcare.” This new specialty would not only include reconstructive and cosmetic surgery but also subspecialties in nursing, dentistry, optometry, and others. By having all groups, no matter their level of training, who want to participate in Restorative Healthcare under one roof, the new specialty could control competition. As long as there are standards, there will always be groups: those who don’t meet the standards, those who want to enter the entity, or those who want to move up a level from within the entity. This new system does not prevent competition. Anyone who has ever worked in a hospital knows that just because everyone's name tag has the same logo, that doesn’t prevent infighting among groups. This proposed alternative specialty of Restorative Healthcare, as described in this article, is doomed to failure because of a top-down organization worse than the current medical board system.

Yes, the system of medical organization is complex and has a lot of regulation, but it has led to tremendous innovation and advanced healthcare. Individuals are living longer with more complex disease states than ever before. Could the system be better, certainly, however, that requires participation by everyone, not a sociobiologic perspective. Plaster surgery has a better chance of avoiding extinction if everyone would be more involved by sitting on hospital credentialing committees, joining state societies to fight restrictive legislation, and joining national organizations to combat intrusions by less qualified groups and not by revisiting high-school biology.
